# Assessment of a Split Homing Based Gene Drive for Efficient Knockout of Multiple Genes

**DOI:** 10.1534/g3.119.400985

**Published:** 2019-12-27

**Authors:** Nikolay P. Kandul, Junru Liu, Anna Buchman, Valentino M. Gantz, Ethan Bier, Omar S. Akbari

**Affiliations:** * Section of Cell and Developmental Biology and; † Tata Institute for Genetics and Society, University of California, San Diego, La Jolla, CA 92093

**Keywords:** CRISPR, Cas9, Homing, split-HGD, *Drosophila melanogaster*, resistance allele

## Abstract

Homing based gene drives (HGD) possess the potential to spread linked cargo genes into natural populations and are poised to revolutionize population control of animals. Given that host encoded genes have been identified that are important for pathogen transmission, targeting these genes using guide RNAs as cargo genes linked to drives may provide a robust method to prevent disease transmission. However, effectiveness of the inclusion of additional guide RNAs that target separate genes has not been thoroughly explored. To test this approach, we generated a split-HGD in *Drosophila melanogaster* that encoded a drive linked effector consisting of a second gRNA engineered to target a separate host-encoded gene, which we term a gRNA-mediated effector (GME). This design enabled us to assess homing and knockout efficiencies of two target genes simultaneously, and also explore the timing and tissue specificity of Cas9 expression on cleavage/homing rates. We demonstrate that inclusion of a GME can result in high efficiency of disruption of both genes during super-Mendelian propagation of split-HGD. Furthermore, both genes were knocked out one generation earlier than expected indicating the robust somatic expression of Cas9 driven by *Drosophila* germline-limited promoters. We also assess the efficiency of ‘shadow drive’ generated by maternally deposited Cas9 protein and accumulation of drive-induced resistance alleles along multiple generations, and discuss design principles of HGD that could mitigate the accumulation of resistance alleles while incorporating a GME.

For standard Mendelian inheritance, any particular allele has a 50% chance in being transmitted to its offspring. While mechanisms of meiosis generally bias selection against violators of Mendel’s rules, there are many examples of naturally occurring selfish genetic elements (SGEs) that succeed in bypassing these rules. These SGEs enhance, or “drive” their transmission into subsequent generations, despite often times being harmful to the harboring individual (*i.e.*, imposing a fitness load). These include, for example, transposable elements (TEs), meiotic drivers, B chromosomes, post segregation killers, heritable microbes, and homing endonuclease genes (Werren *et al.* 1988; Burt and Trivers 2006; Werren 2011; McLaughlin and Malik 2017). Drawing inspiration from these natural systems, strategies for exploiting drive to alter the genetics of wild pest populations have been proposed (Werren *et al.* 1988; Burt, 2003; Burt and Trivers 2006; Werren 2011; Esvelt *et al.* 2014; Champer *et al.* 2016; McLaughlin and Malik 2017), and some have even been experimentally tested in the laboratory, however none have been implemented in the field. For those tested in the laboratory, some examples include synthetic *Medea* elements (Chen *et al.* 2007; Akbari *et al.* 2014; Buchman *et al.* 2018a), engineered underdominance systems (Akbari *et al.* 2013; Buchman *et al.* 2018b), and those whose development was accelerated by the CRISPR revolution (Jinek *et al.* 2012; Cong *et al.* 2013; Mali *et al.* 2013) including toxin-antidote based systems (Oberhofer *et al.* 2019), and homing based gene drive systems (HGDs) (Esvelt *et al.* 2014; Gantz and Bier 2016; Champer *et al.* 2016; Marshall and Akbari 2018).

HGDs are perhaps the furthest along in development, and have already been tested in a broad range range of organisms spanning bacteria, yeast, insects, and mammals (Windbichler *et al.* 2011; Gantz and Bier 2015; DiCarlo *et al.* 2015; Gantz *et al.* 2015; Hammond *et al.* 2016, 2018; Champer *et al.* 2017, 2018; KaramiNejadRanjbar *et al.* 2018; Kyrou *et al.* 2018; Yan and Finnigan 2018; Li *et al.* 2019; Grunwald *et al., *2019, Valderrama *et al.*, 2019). They function by encoding the Cas9 endonuclease and an independently expressed guide RNA (gRNA) responsible for mediating DNA/RNA base pairing and cleavage at a predetermined site (Esvelt *et al.* 2014; Gantz and Bier 2015, 2016; Champer *et al.* 2016; Marshall and Akbari 2018). When the HGD is positioned within its target site in a heterozygote, double stranded DNA breaks (DSBs) on the opposite chromosome can result in the drive allele being used as a template (*i.e.*, donor chromosome) for DNA repair mediated by homologous recombination. This can result in copying, or “homing,” of the HGD into the broken chromosome (*i.e.*, receiver chromosome), thereby converting heterozygotes to homozygotes in the germline, which can bias Mendelian inheritance ratios and result in an increase in HGD frequency in a population.

Given the recent progress toward developing HGDs in pest species such as mosquitoes (Gantz *et al.* 2015; Hammond *et al.* 2016, 2018; Kyrou *et al.* 2018; Li *et al.* 2019), there is significant enthusiasm regarding their potential use to control wild populations. For example, given the enormous burden mosquitoes pose on humans, the release of HGDs linked with effector genes inhibiting mosquito pathogen transmission (Isaacs *et al.* 2011; Jupatanakul *et al.* 2017; Buchman *et al.* 2019a; b) may lead to replacement of disease-susceptible mosquitoes with disease-resistant counterparts resulting in reduced pathogen transmission (*i.e.*, population modification drive). Alternatively, HGDs targeting genes affecting the fitness of female mosquitoes could also spread, resulting in gradual population declines and potentially even elimination (*i.e.*, population suppression drive) (Windbichler *et al.* 2008, 2011; Kyrou *et al.* 2018). Given these features, both modification and suppression drives possess the potential to transform mosquito population control measures (Burt 2003; Esvelt *et al.* 2014; Gantz and Bier 2016; Champer *et al.* 2016), and therefore have excited significant ongoing discussions involving their potential usage, regulation, safety, ethics and governance (Oye *et al.* 2014; Akbari *et al.* 2015; National Academies of Sciences, Engineering, and Medicine *et al.* 2016; Adelman *et al.* 2017). Despite these exciting developments however, the elephant in the room persists - can a gene drive actually work in the wild? There are a number of open questions looming as to the efficiency of HGDs. For example, can a drive spread to fixation in the wild? Will it simply breakdown due to resistance? Will the linked anti-pathogen effector work efficiently given the expected diversity of parasites/virus genomes found in the wild? Can the pathogen evolve to become resistant to the anti-pathogen effector and perhaps even become more virulent (Marshall *et al.* 2019)? These are just a minority of legitimate concerns regarding the potential use of a gene drive that would need to be resolved prior to any release.

While many questions loom, there has been some effort to resolve these concerns safely in the lab. For example, with regard to the HGD breakdown due to resistance, multiple studies have explored design criteria attempting to suppress the effects of resistance alleles on drive propagation. For example, some studies have had some success using germline-restricted promoters to express Cas9 increasing rates of HDR, resulting in increased homing rates, as opposed to error-prone pathways such as non-homologous end joining (NHEJ) which results in the generation of resistance alleles (Hammond *et al.* 2018; Champer *et al.* 2018). Other studies have described (Esvelt *et al.* 2014; Champer *et al.* 2016; Marshall *et al.* 2017) and tested (Champer *et al.* 2018, 2019a; b; Oberhofer *et al.* 2018) multiplexed gRNAs in drives resulting in moderate increases in drive efficacy. While others have had some success targeting highly conserved recessive fertility/viability genes whose homozygous mutants are inviable, or cannot reproduce, and therefore are expected to not affect the spread of HGDs (Hammond *et al.* 2016; KaramiNejadRanjbar *et al.* 2018; Kyrou *et al.* 2018; Oberhofer *et al.* 2018). However, despite these efforts, resistance alleles are still problematic, leaving open the question as to what is the best method to prevent their generation.

Here, to further explore this paramount issue of resistance to HGD we use *Drosophila melanogaster* as our model. We use a genetic safeguarded split-drive design as a safety feature and also encode a linked effector to the drive. This effector consisted of a second gRNA engineered to target a separate host encoded gene which we term a gRNA-mediated effector (GME) (Figure 1). Given that there are many host-encoded genes that are important for pathogen transmission (Cheng *et al.* 2016; Dong *et al.* 2018), one potential application of a HGD is to incorporate a cargo GME that targets a host encoded factor that is important for some aspect of pathogen transmission. If the GME is effective, then disruption of its target in the population should in principle occur as the drive spreads, thereby immunizing that population from pathogen transmission. Therefore, encoding a GME in a drive may be a useful feature going forward and worth further exploring. As a proof of concept to test the efficiency of a HGD linked GME, we designed both the drive and effector to target phenotypic genes which resulted in easily scorable recessive viable phenotypes. This novel drive architecture enabled us to test many germline Cas9 expressing promoters, while simultaneously measuring homing and cleavage efficiencies in both the germline and soma for both target genes over successive generations. While homing rates were modest, cleavage rates were high. For example, we determined that we can reproducibly achieve complete penetrance of somatic mosaic phenotypes for both target genes with up to 100% efficiency stemming from a combination of Cas9 maternal deposition and somatic expression. However, despite the robust cleavage efficiencies and impressive efficacy of the HGD linked GME, drive resistance alleles were still generated which would hinder spread. Given these results, alternative design principles are proposed that could potentially mitigate these issues while also incorporating a drive linked GME.

**Figure 1:**
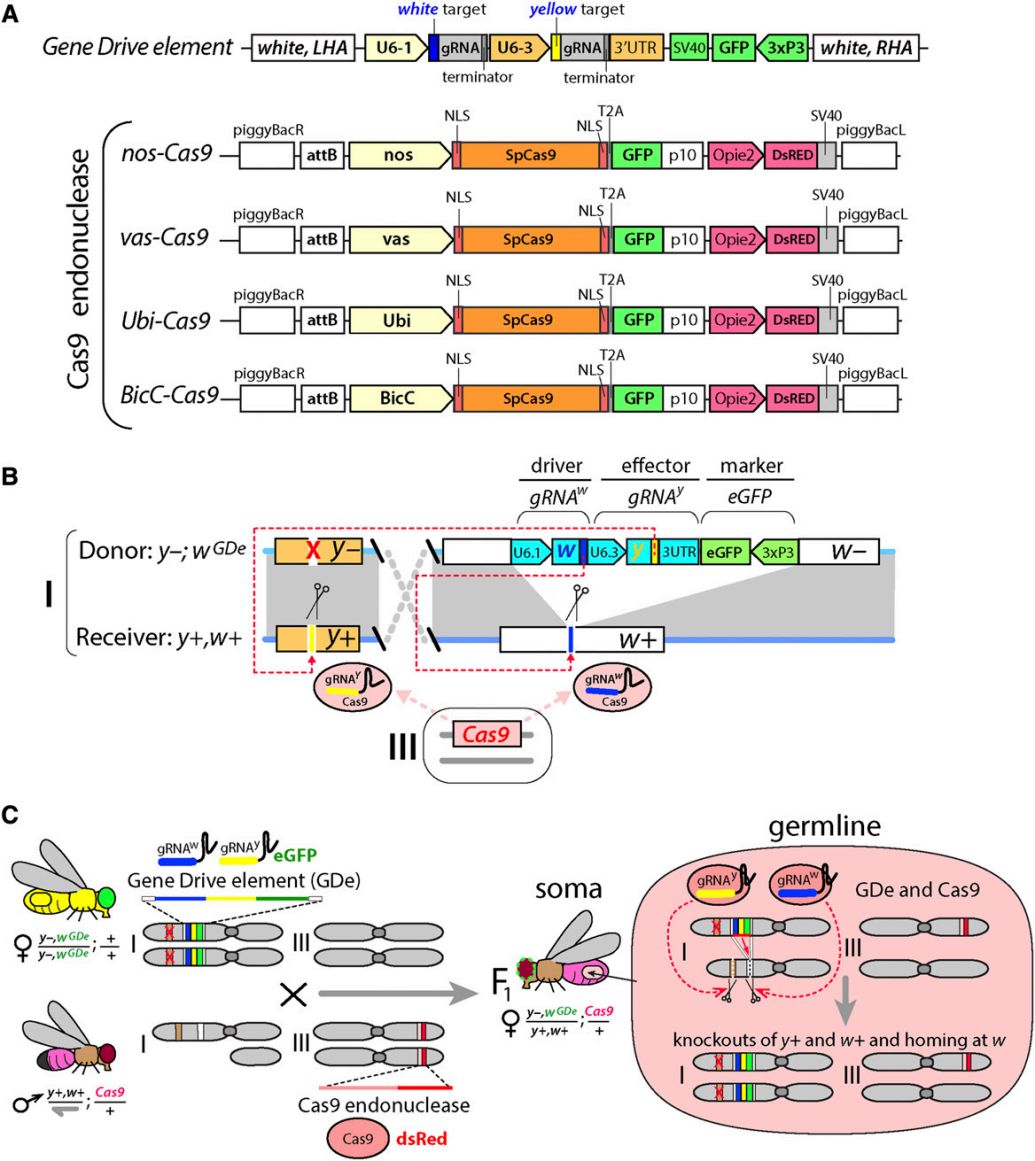
Development of the CRISPR/Cas9-mediated split-drive system. The homing gene drive (HGD) system was split into two components: *Gene Drive* element (*GDe*) and *Cas9* endonuclease (Cas9). (A) Schematic maps (not to scale) of genetic constructs used to assemble split-HGD systems. The GDe contains two guide RNAs (gRNAs) targeting the DNA cleavage at *white* and *yellow* loci, and an eye-specific marker (*3xP3-GFP)* all surrounded by Left and Right Homology Arms (LHA and RHA) complementary to the *white* cut site. Four Cas9 constructs expressing *SpCas9* (*Cas9*) in early germline cells with *nanos* (*nos*) and *vasa* (*vas*) promoters, in late germ cells with *Bicaudal C* (*BicC*) promoter, and in both germ and somatic cells with *Ubiquitin 63E* (*Ubi*) promoter carried the *eGFP* linked to the coding sequence of Cas9 via a self-cleaving *T2A* sequence and a body specific marker of transgenesis (*Opie2-DsRed*). (B) *GDe* was site-specifically inserted at *white* locus on the 1^st^ chromosome (*i.e.*, X chromosome) in *Drosophila* via HDR-mediated integration, *w^GDe^*. The Cas9 constructs were inserted at the same site on the 3^rd^ chromosome using φC31-mediated integration. In the presence of Cas9, *GDe* direct cleavage at both *w+* and *y+* loci and can home at *white* locus from the *w^GDe^* donor allele into the *w+* receiver allele via HDR in heterozygotes. (C) The genetic cross between the *GDe* and *Cas9* homozygous lines generates *trans*-heterozygous *y–,w^GDe^/y+,w+*; *Cas9/+* females. The germline Cas9 expression is expected to limit the activity of the split-drive system, *y+* and *w+* knockouts and *w^GDe^* homing, to germ cells of the *y–,w^GDe^/y+,w+*; *Cas9/+* females. The *w^GDe^* allele cannot home in *Drosophila* males, because they have only one X chromosome, aka. hemizygous.

## Materials and Methods

### Design and assembly of constructs

The genetic assembly of the Gene Drive element (*GDe*) with two gRNAs and 3xP3-eGFP (Figure 1A) was previously described to generate a split *trans*-complementing Gene Drive system (Lopez del Amo *et al.* 2019). The assembly of BicC*-Cas9* construct followed the same steps previously described for the other three Cas9 lines: nos*-Cas9*, vas*-Cas9*, and *Ubi-Cas9* (Kandul *et al.* 2019). The 2831 bases upstream of BicC-RA’s start codon (*Bicaudal C*, CG4824) was PCR amplified with CGACGGTCACGGCGGGCATGTCGACGCGGCCGCATAATTATATAATAATAAACTGCATGC (BicC-F) and TCCGTCGTGGTCCTTATAGTCCATGTTTAAACTGTGGAATTCGGATGATGATGATGATC (BicC-R) from *Drosophila melanogaster* genome, and enzymatically assembled (Gibson *et al.* 2009) into *Ubi-Cas9* plasmid (addgene #112686) (Kandul *et al.* 2019) digested with NotI and XhoI.

### Fly genetics and imaging

Flies were maintained under standard conditions at 25°. Embryo injections were carried at Rainbow Transgenic Flies, Inc. (http://www.rainbowgene.com). The BicC*-Cas9* construct was inserted at the PBac{y+-attP-3B}KV00033 on the 3^rd^ chromosome (Bloomington #9750) with φC31-mediated integration (Groth 2004). Transgenic flies were balanced with Df(3L)R/TM6C,cu^1^,Sb^1^,Tb^1^ (Bloomington #57) and CxD,ry^BM^/TM3,Sb^1^,Ser^1^ (Bloomington #1704) in the *w+* genetic background.

**Figure 2:**
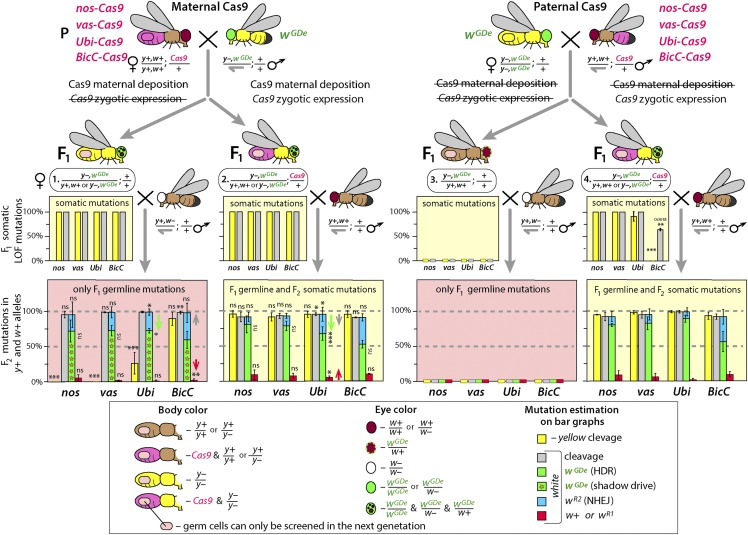
Multiple Cas9 promoters induce double-gene knockouts in somatic tissues independently of maternal or paternal inheritance of Cas9. Each tested Cas9 promoter, including previously characterized germline-limited *nos*, *vas*, and *BicC* promoters, supported Cas9 expression in F_1_ somatic tissues and resulted in *white* and *yellow* loss-of-function (LOF) mutations in the F_1_ progeny. Furthermore, maternal deposition of Cas9 protein alone was sufficient to generate F_1_ somatic *white* and *yellow* LOF mutations as well as induce both homing (*w^GDe^*) and formation of resistance alleles (*w^R2^*) in *w+* alleles of their germ cells (F_1_ ♀ #1). Paternal deposition of Cas9 protein did not induce mutations in somatic or germ cells (F_1_ ♀ #3). Notably, while 100% F_1_ parents had *yellow* LOF somatic mutations with each tested *Cas9* line, only *Ubi* and *BicC* promoters deposited Cas9 protein sufficient to induce *yellow* LOF mutations in some germ cells (F_1_ ♀ #1). Zygotic expression of *Cas9* under *nos*, *vas*, and *Ubi* promoters induced *white* and *yellow* LOF mutations in 100% *trans*-heterozygous females, while zygotic expression of BicC*-Cas9* caused only *white* LOF mutation in 64.3% ± 2.6% of *trans*-heterozygous females (F_1_ ♀ #4). Rates of homing and resistance alleles were not significantly different among two types of *trans*-heterozygous (F_1_ ♀ #2 and #4) and heterozygous (♀ #1) females with maternally deposited Cas9. Only maternal deposition of Cas9 under *Ubi* promoter negatively affected homing rates (green arrows) in germ cells. Bar plots show the average ± SD over at least three biological replicate crosses. Statistical significance was estimated using a *t*-test with equal variance. (*P* ≥ 0.05^ns^, *P* < 0.05*, *P* < 0.01**, and *P* < 0.001***).

To assess the cleavage rates and homing efficiencies of the split-drive system, we genetically crossed the *GDe* line to four different *Cas9* lines in both directions. Two types of F_1_ *trans*-heterozygous *y–,w^GDe^(eGFP)/y+,w+*; *Cas9(RFP)/+* females carrying either maternal or paternal *Cas9* (F_1_ **♀** #2 or **♀** #4, respectively) and the F_1_ heterozygous *y–,w^GDe^(eGFP)/y+,w+* females with either maternally or paternally deposited Cas9 protein were generated (F_1_ **♀** #1 or **♀** #3, respectively; Figure 2). Their *yellow* and *white* LOF mutations and transgene markers were scored. To explore whether *yellow* and *white* loci were also mutated in the germ cells of the F_1_ *trans*-heterozygous and heterozygous females, we genetically crossed them to *w+,y+* and *w–,y+* males, respectively, and examined their F_2_ progeny. LOF *yellow* mutations were scored only in male progeny that inherited their single X chromosome from mothers. To explore the behavior of resistance alleles over multiple generations, the F_2_ *trans*-heterozygous and heterozygous virgin female (**♀** #6 or **♀** #5, respectively) progeny of F_1_ **♀** #2 were also collected, and genetic crosses and phenotype scoring were repeated for an additional generation, F_3_. The above crossing schemes are depicted in Figure 2. To generate means and standard deviations for statistical comparisons, each genetic cross was set up in triplicate using 10♂ and 10♀ flies for each replicate cross. Cleavage and homing frequencies are presented as percentages of *y+* and *w+* alleles in heterozygous females, aka. they normalized to 50% (Table S1).

Flies were examined, scored, and imaged on the Leica M165FC fluorescent stereo microscope equipped with the Leica DMC2900 camera. To analyze Cas9 expression in ovaries of four homozygous *Cas9* lines, their ovaries were dissected in PBS buffer, examined, and imaged utilizing the same settings. The eGFP fluorescence was used as a proxy of Cas9 expression, since it was tagged to *Cas9* transgene via a *T2A* sequence (Figure S1).

### Genotyping loci targeted with gRNAs

To explore the molecular changes that caused LOF and in-frame functional mutations in *yellow* and *white* loci, we PCR amplified the genomic regions containing target sites for *gRNA^w^* and *gRNA^y^*: GGCGATACTTGGATGCCCTGCGG and GGTTTTGGACACTGGAACCGTGG, respectively. Single-fly genomic DNA preps were prepared by homogenizing a fly in 30µl of a freshly prepared squishing buffer (10mM Tris-Cl pH 8.0, 1mM EDTA, 25mM NaCL, 200 μg/mL Proteinase K), incubating at 37° for 35 min, and heating at 95° for 2 min. 2 µl of genomic DNA was used as template in a 40 µL PCR reaction with LongAmp Taq DNA Polymerase (NEB). The 415bp PCR fragment of *white* target was amplified with CGTTAGGGAGCCGATAAAGAGGTCATCC (w.sF) and AAGAACGGTGAGTTTCTATTCGCAGTCGG (w.sR); and CACTCTGACCTATATAAACATGGACCGCAGTTTG (y.sF) and CCAATTCATCGGCAAAATAGGCATATGCAT (y.sR) primers were used to amplify the 375bp PCR fragment of *yellow*. PCR aplicons were purified using QIAquick PCR purification kit (QIAGEN), and sequenced in both directions with Sanger method at Source BioScience. To characterize molecular changes at the targeted sites, sequence AB1 files were aligned against the corresponding reference sequences in SnapGene 4.

### Statistical analysis

**Figure 3:**
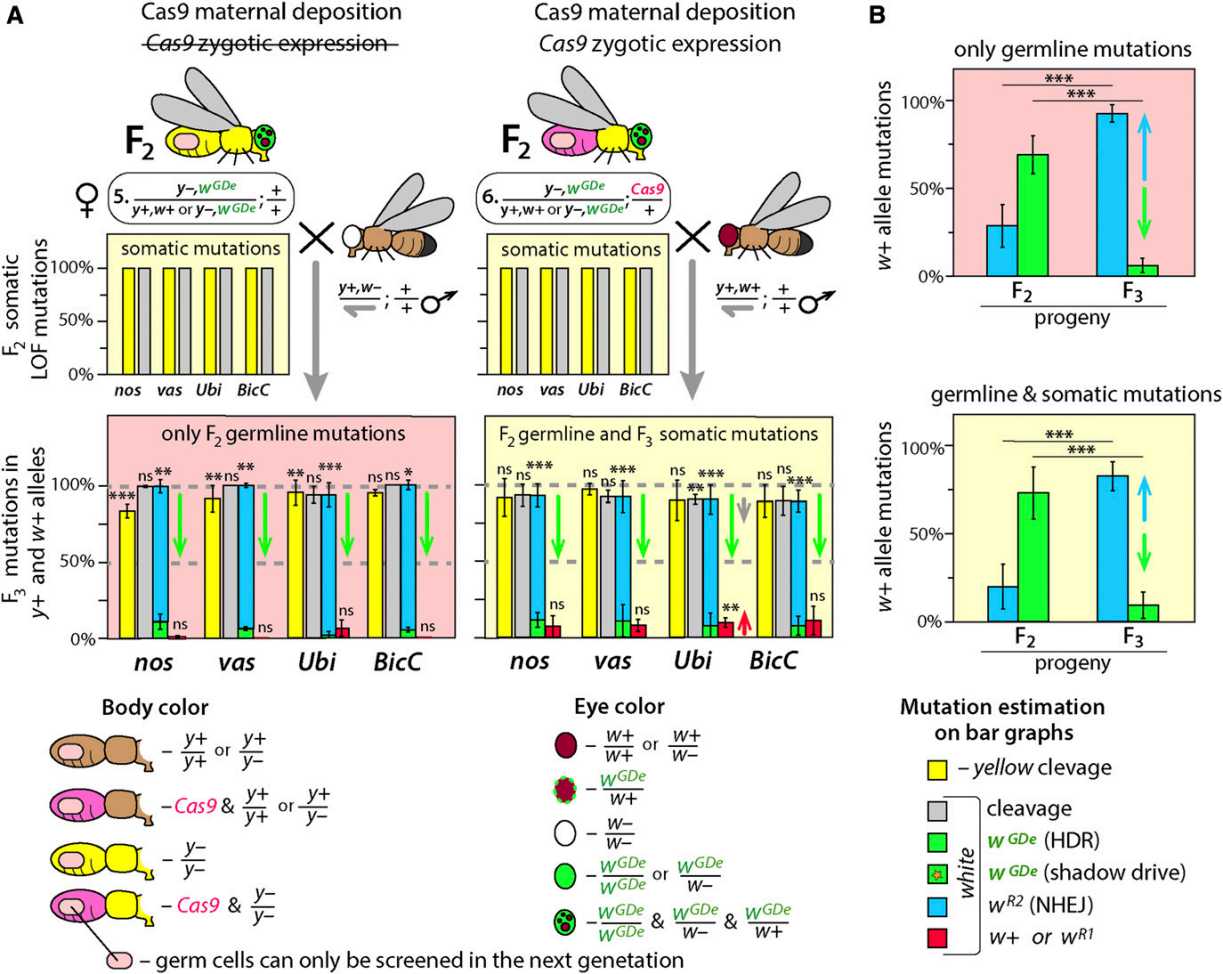
Resistance alleles accumulates over subsequent generations and restricts homing. Resistance alleles are expected to be immune to the further cleavage by the same Cas9/gRNA system and if their carrier is fertile can propagate at the expense of homing. (A) To explore this phenomenon, F_2_ ♀ #5 and F_2_ ♀ #6 collected among progeny of F_1_ ♀ #2 were genetically crossed with *w*– and *w+* males, respectively, and their F_3_ progeny were scored. While the cleavage rate in F_2_ germ cells decreased only in F_2_ ♀ #6 with *Ubi-Cas9* (red arrow) likely due to the rise of functional w*^R1^* alleles, the homing frequency fell significantly for each tested split-drive system with and without *Cas9* gene (green arrows). The fall of homing rate was accompanied by the accumulation of the *w^R2^* alleles. (B) Accumulation of *w^R2^* alleles resistant to cleavage by Cas9/gRNA^w^ restricted homing of GDe. Frequencies of homing and resistance alleles were averaged for all tested promoters and presented separately for progeny of heterozygous and *trans*-heterozygous females, F_2_ ♀ #5 and F_2_ ♀ #6, respectively. Resistance allele frequency increased from 28.5% or 19.9% to 92.6% or 82.6%, respectively, between F_2_ and F_3_ (blue arrows) and caused the dramatic decline in homing from 69.0% or 73.0% to 6.1% or 9.2%, respectively (green arrows). Notably, scoring of *w^R2^* alleles in *w*– recessive background resulted in the higher estimation of *white* LOF mutations alleles, since *w^R2^* alleles were complemented by *w+* alleles inherited from wild type males. Bar plots show the average ± SD over at least three biological replicate crosses. Statistical significance was estimated using a *t*-test with equal variance. (*P* ≥ 0.05^ns^, *P* < 0.05*, *P* < 0.01**, and *P* < 0.001***).

Statistical analysis was performed in JMP 8.0.2 by SAS Institute Inc. At least three biological replicates were used to generate statistical means for comparisons. To estimate the effect of Cas9 maternal deposition on homing efficiency, rates of cleavage, homing, and resistance allele formation in F_1_ **♀** #4 with paternal Cas9 were compared to the corresponding values in F_1_ **♀** #1 and **♀** #2 with maternally deposited Cas9 protein (Figure 2). To assess the significance of resistance allele accumulation and homing rate decline between F_2_ and F_3_ generations, rates of cleavage, homing, and resistance alleles in F_2_ **♀** #5 and F_2_ **♀** #6 (Figure 3A) were compared to the corresponding values in F_1_ **♀** #1 and F_1_ **♀** #2, respectively (Figure 2). *P* values were calculated for a two-sample Student’s *t*-test with equal variance.

### Gene drive safety measures

All crosses using gene drives genetics were performed in accordance to an Institutional Biosafety Committee-approved protocol from UCSD in which full gene-drive experiments are performed in a high-security ACL2 barrier facility and split drive experiments are performed in an ACL1 insectary in plastic vials that are autoclaved prior to being discarded in accord with currently suggested guidelines for laboratory confinement of gene-drive systems (Akbari *et al.* 2015; National Academies of Sciences, Engineering, and Medicine *et al.* 2016).

### Ethical conduct of research

We have complied with all relevant ethical regulations for animal testing and research and conformed to the UCSD institutionally approved biological use authorization protocol (BUA #R2401).

### Data availability

All data that are represented fully within the tables and figures. The *nos*-, *vas*-, *Ubi-Cas9* plasmids and the corresponding fly lines are deposited at Bloomington Drosophila Stock Center (#79004 – #79006) and AddGene.org (#112685 – #112687), respectively. The BicC*-Cas9* and *GDe* plasmids and fly lines will be made available upon request. Supplemental material available at figshare: https://doi.org/10.25387/g3.11449542.

## Results

### Design of split-HGD encoding two gRNAs

To assess the feasibility and efficiency of utilizing a HGD to bias transmission while also expressing a GME, we designed a HGD that expressed two gRNAs (Lopez del Amo *et al.* 2019). The homing component of the split-HGD system, referred herein as a Gene Drive element (GDe), encodes a gRNA targeting *white* (*gRNA^w^*, driver gRNA), a separate cargo GME targeting *yellow* (*gRNA^y^*, effector), a *3xP3-eGFP* dominant marker, all together flanked by 1kb homology arms from the *white* target locus to direct targeted HDR mediated integration (Figure 1A). The *GDe* was integrated at the *white* locus (*w^GDe^*) in *D. melanogaster* via HDR. In the presence of Cas9, the *GDe* directs cleavage at both *white* and *yellow*, both X-linked loci, and is also capable of homing into the *white* locus (Figure 1B-C). Importantly, in *D. melanogaster* homozygous loss-of-function (LOF) mutants of both *white* and *yellow* are viable and fertile with scorable recessive LOF phenotypes in the eye and body, respectively, enabling cleavage events to be directly quantified over successive generations. Additionally, males have only one X chromosome, and are therefore hemizygous for *white* and *yellow*, restricting the quantification of homing to heterozygous females (y–,*w^GDe^/y+,w+*, Figure 1C).

### High penetrance of F_1_ somatic mutations generated by Cas9 through both maternal deposition and zygotic expression

We explored the effects of tissue specificity and timing of Cas9 expression on cleavage and homing in the germline by using four separate promoters with distinct expression profiles to express Cas9-T2A-GFP: nanos* (nos)* (Van Doren *et al.* 1998) and vasa* (vas)* promoters known for early germline-limited expression (Hay *et al.* 1988; Van Doren *et al.* 1998; Sano *et al.* 2002); *Bicaudal C* (BicC*)* promoter supporting later germline-limited expression (Saffman *et al.* 1998); and *Ubiquitin 63E* (*Ubi*) promoter with strong expression in both somatic and germ cells (Preston *et al.* 2006; Akbari *et al.* 2009). We controlled for variation in expression due to position effect (PE), by integrating each Cas9 construct (Figure 1A) into the same site on the 3^rd^ chromosome using φC31-mediated integration (Groth 2004). We confirmed germline expression by imaging the expression of a self-cleaving T2A-eGFP tag attached to the coding sequence of Cas9, and each promoter robustly expressed GFP in the ovaries (Lowest nos*-Cas9* < vas*-Cas9* < *Ubi-Cas9* < BicC*-Cas9* Highest) (Figure S1).

We quantified cleavage efficiencies by performing bi-directional crosses between hemizygous or homozygous GDe lines mated to heterozygous Cas9 lines (Figure 2). From these crosses we determined that maternally deposited Cas9 protein is sufficient to induce both *yellow* and *white* somatic LOF mutations in F_1_ females heterozygous for the *GDe* both in presence (♀# 2; *y*–,*w^GDe^/y+,w+*; Cas9/+) and in the absence (♀ # 1; *y*–,*w^GDe^/y+,w+*; Figure 2) of *Cas9* gene inheritance. To determine whether zygotic expression of *Cas9* can also induce somatic mutations, we scored *white* and *yellow* LOF somatic mutations in F_1_ *trans*-heterozygous females inheriting *Cas9* exclusively from their fathers (*i.e.*, paternal *Cas9*). Unexpectedly, F_1_ *trans*-heterozygous female progeny inheriting Cas9 as a gene (♀#4; y–,*w^GDe^/y+,w+*; Cas9/+; Figure 2) from their fathers had mutations in both *white* and *yellow* with varying frequencies depending on which promoter drove Cas9 expression. For example, nos*-Cas9* and vas*-Cas9* – induced 100% *white* and *yellow* LOF somatic mutations in F_1_ *trans*-heterozygous females, while *Ubi-Cas9* resulted in 100% of *white* and 91.3% ± 9.7% of *yellow* LOF somatic mutations, and BicC*-Cas9* resulted in only *white* LOF mutations in 64.3% ± 2.6% of the F_1_ y–,*w^GDe^/y+,w+*; BicC*-Cas9*/+ progeny (Figure 2). Interestingly however, 100% of F_1_ heterozygous female progeny from the same fathers that did not inherit Cas9 as a gene (♀#3; y–,*w^GDe^/y+,w+*; +/+; Figure 2) had wild type (*wt)* phenotypes, for both *white* (red eyes) and *yellow* (brown body), presumably resulting from lack of sufficient Cas9 protein deposited paternally to induce mutations in the zygote (Table S1). Taken together these data indicate that the Cas9 promoters tested here are active both maternally and zygotically and can promote very high cleavage efficiency in somatic cells.

We assessed whether the *yellow* and *white* alleles were mutated by maternally deposited Cas9 in germ cells of F_1_ *y–,w^GDe^/y+,w+* females by mating these females to *y+,w*– males and scored recessive *yellow* phenotypes in resulting F_2_ male progeny (*y*–) and recessive *white* phenotypes in resulting F_2_ male and female progeny (*w–/w*–). We found that maternally deposited Cas9 protein expressed under *nos* and *vas* promoters did not induce *yellow* LOF mutations in germ cells of F_1_ females, while expression from *Ubi* and *BicC* promoters resulted in 26% ± 15% and 89.4% ± 9.4% of *yellow* alleles being mutated in germ cells of F_1_ females (Figure 2), respectively, perhaps due to a stronger maternal deposition of Cas9 protein by these promoters (Figure S1) combined with possible preferential gRNA loading by Cas9. Despite the lack of LOF germline mutations in *yellow* by *nos* and *vas*, every tested Cas9 line provided a sufficient amount of maternally deposited Cas9 protein to knockout the *white* allele in 94.9% ± 4.5–98.8% ± 1.1% of F_1_ germ cells (measured in F_2_ progeny; Figure 2, Table S1). We explored whether the *w+* alleles (1.2–5.1%) were cut by Cas9, and perhaps repaired into cleavage resistance alleles, by perfoming Sanger sequencing of PCR amplicons of the *white* target locus from individual male flies. Each tested F_2_ male with red eyes (w+) indeed had a *wt w+* allele, and we did not find any *white* in-frame functional resistance alleles in F_1_ germ cells suggesting that these alleles likely remained uncut in the germline.

### Maternally deposited Cas9 is sufficient to induce homing of GDe in germ cells

The Cas9/gRNA^w^-induced DSBs at *white* locus can be repaired either by HDR resulting in homing of the *GDe* (*w^GDe^/w^GDe^*) or NHEJ incorporating *indel* mutations that can render the target locus unrecognizable by the Cas9/gRNA^w^ machinery, and when these mutations occur in germ cells they are referred to as resistance alleles (*w^R^)*: here LOF and in-frame functional resistance alleles are referred as *w^R2^* and *w^R1^*, respectively (Figure S2). To directly estimate the frequency of *w^GDe^* homing and *w^R^* generation in the absence of additional somatic mutations resulting from zygotic expression of *Cas9*, we analyzed *white* phenotypes in the F_2_ progeny of the F_1_ *w^GDe^/w+* females with maternally deposited Cas9 in a *w*– recessive mutant background (Figure 2). Every tested Cas9 promoter provided a sufficient amount of maternally deposited Cas9 in the F_1_ germ cells to enable the conversion of 59–72% of *w+* alleles into *w^GDe^* (*i.e.*, homing of *GDe)* in *y*–,*w^GDe^/y+,w+* females. This conversion which occurs in the presence of Cas9 protein, but absence of inheritance of the *Cas9* gene, was previously noted and termed “shadow drive” (Guichard *et al.* 2019). The remaining DSBs at *w+* alleles were repaired by NHEJ and generated around 38–23% *w^R2^* alleles (Figure 2). To explore molecular changes at *white* locus, we PCR amplified and Sanger sequenced *w^R2^* alleles from individual F_2_ male progeny and identified *indels* localized at the *white* cut site in each sequenced male (Figure S3A). The maternally deposited Cas9 by *BicC* promoter resulted in the lowest homing and the highest resistance allele rates (59.3% ± 12.3% and 38.7% ± 13.7%, respectively), though no significant difference was identified between *BicC* and other Cas9 promoters. Nevertheless, each tested promoter supplied Cas9 protein via mothers to the progeny that enabled shadow drive, thus resulting in super-Mendelian propagation of *w^GDe^* to their grandchildren.

### Maternal deposition of Cas9 protein reduces the homing efficiency

Maternally deposited Cas9 can induce *white* cleavage and repair mediated by NHEJ as opposed to HDR in mitotically dividing germ cells which can result in a bias toward generating resistance alleles (*w^R2^* and *w^R1^*) at the expense of homing *w^GDe^* (Lopez del Amo *et al.*, 2019). To explore this effect, we compared homing rates between F_1_ *trans*-heterozygous females that inherited Cas9 either maternally (♀#2; *y*–,*w^GDe^/y+,w+*; Cas9/+) or paternally (♀#4; *y*–,*w^GDe^/y+,w+*; Cas9/+; Figure 2). For nos*-Cas9*, vas*-Cas9*, and BicC*-Cas9*, maternal deposition of Cas9 did not result in a significant bias in homing efficiencies. However, for *Ubi-Cas9* homing rates were significantly lower (67%) in the *trans*-heterozygous females that inherited *Cas9* maternally (♀#2; *w^GDe^/w+*; *Ubi-Cas9/+*) as compared to 88% for *trans*-heterozygous females inheriting *Ubi-Cas9* paternally (♀#4; *w^GDe^/w+*; *Ubi-Cas9/+*). In addition to the lower homing rates for *Ubi-Cas9*, the rate of *w^R2^* alleles was significantly higher with maternally deposited *Ubi-Cas9* as compared to paternally deposited *Ubi-Cas9*: 9.9% ± 5.7% *vs.* 27.3% ± 10.0%, *P* > 0.025 or 26.5% ± 4.4%, *P* > 0.029, respectively (Figure 2). Taken together, these results suggest that high levels of maternal deposition of Cas9 protein into developing oocytes can result in *white* cleavage in mitotic cells, prior to developmental stages where efficient HDR repair occurs, therefore leading to a higher frequency of *w^R^* events.

### Resistance alleles accumulate between F_2_ and F_3_ generations

Resistance alleles generated in germ cells are immune to subsequent cleavage by the Cas9/gRNA^w^ complex. Drosophila white and *yellow* LOF homozygotes are viable and fertile, as a result, the frequency of resistance alleles can potentially increase from generation to generation. We explored this possibility by crossing F_2_ *trans*-heterozygous females (♀ #6, *w^GDe^/w+*; *Ubi-Cas9/+*; Figure 3A) to *wt (y+*,w+) males, and scored their F_3_ progeny for *yellow* and *white* phenotypes, as well as for inheritance of the *GDe*. Indeed, the frequency of *white* LOF mutations (*w^R2^*) increased significantly between F_2_ and F_3_ progenies for each Cas9 promoter: 11.2% ± 6.2% *vs.* 81.7% ± 7.5% for nos*-Cas9*; 13.2% ± 5.6% *vs.* 82.4% ± 10.4% for vas*-Cas9*; 18.6% ± 12.0% *vs.* 84.6% ± 9.5% for *Ubi-Cas9*; and 36.7% ± 7.5% *vs.* 81.6% ± 7.1% for BicC-Cas9, *P* > 0.0001, respectively. This increased frequency of generating *w^R2^* alleles negatively affected the homing rate, which dropped between F_2_ and F_3_ generations: from 80.0% ± 7.7% to 11.3% ± 4.8% for nos*-Cas9*; from 80.2% ± 7.4% to 10.8% ± 10.4% for vas*-Cas9*; from 78.0% ± 13.2% to 7.4% ± 8.4% for *Ubi-Cas9*; and from 53.9% ± 9.8% to 7.6% ± 6.2% for BicC*-Cas9* (♀ #6, *w^GDe^/w+*; *Ubi-Cas9/+*; Figure 3A). To avoid any ambiguity caused by somatic expression of Cas9, the same analysis was repeated with the F_2_ heterozygous females carrying maternally deposited Cas9 protein but lacking the *Cas9* gene resulting in similar conclusions (♀ #5, *y–,w^GDe^/y+,w+*; Figure 3A). We assessed the accumulation of resistance alleles by comparing the mean frequencies of homing and resistance alleles between F_2_ and F_3_ generations. The frequency of resistance alleles rose from 28.5% ± 12.2% to 92.6% ± 5.0% in heterozygous females or from 19.9% ± 12.8% to 82.6% ± 8.2% *in trans*-heterozygous females, and decreased the homing rate from 69.0% ± 10.8% to 6.1% ± 4.2% and from 73.0% ± 14.6% to 9.2% ± 7.5%, respectively (*P* > 0.0001, Figure 3B). As expected, the frequency of LOF resistance alleles at *white* locus (*w^R1^*) also increased from F_2_ to F_3_ generations and further restricted homing of the *GDe*. The frequency of in-frame functional *white* and *yellow* mutations (*w^R1^* and *y^R1^*) could also increase in the F_3_ progeny, but unfortunately this effect could not be directly estimated. The frequency of cleavage at *white* significantly decreased in the F_3_ progeny of F_2_ *y–,w^GDe^/y+,w+*; *Ubi-Cas9/+* females, and could be explained by the increase of *w^R1^* allele rate that were indistinguishable from *w+* alleles phenotypically: from 3.4% ± 2.6% in F_2_ to 9.7% ± 3.1% in F_3_, *P* > 0.004 (Figures 2, 3A). We tested this hypothesis by Sanger sequencing F_3_ *wt* males with red eyes and brown bodies, and identified in-frame *indels* and substitutions in the majority of tested males for each Cas9 promoter (*w^R1^* and *y^R1^* alleles, Figure S3). Therefore, many germ cells of F_2_ *trans*-heterozygous and heterozygous with maternally deposited Cas9 females had *indel* mutations in the *white* and *yellow* loci (*y–,w^GDe^/y^R^,w^R^*) that were indeed resistant to further cleavage by Cas9/gRNA^w^ and Cas9/gRNA^y^, respectively.

## Discussion

Homing based gene drives require efficient cleavage and copying in the germline in order to bias their transmission and are therefore sensitive to both existing and induced target sequence variation. In fact, the NHEJ-mediated generation of resistance alleles in germ cells was previously identified as the major force opposing the spread of HGD into populations (Gantz *et al.* 2015; Champer *et al.* 2017; Hammond *et al.* 2017; Oberhofer *et al.* 2018). Here, we used a split-drive design to further explore the effect of timing and location of Cas9 expression on both homing and resistance allele formation. This experimental design enabled us to separate effects of somatic expression from maternal deposition of Cas9 on the *GDe* inheritance and mutagenesis of a targeted gene. Additionally, we linked a GME to the GDe to measure the efficacy of the knockout of an additional gene, *yellow*. Using this approach, we were able to draw several conclusions, including; i) in addition to germline expression, each tested Cas9 promoter (*nos*, *vas*, *BicC*, *Ubi*) directs significant expression in somatic tissues; ii) the maternal protein deposition or gene expression of Cas9 is sufficient for homing (shadow drive) in germ cells; iii) paternal Cas9 protein deposition in the sperm is insufficient for the mutagenesis of a target gene; iv) drive-induced resistance alleles accumulate over generations and are predicted to restrict the spread of the drive; and v) expression of a drive mediating gRNA in addition to a linked GME can result in 100% penetrance of both scorable LOF phenotypes. Below we discuss these conclusions further and also propose novel drive architectures to potentially overcome these issues.

### Somatic expression of Cas9 results in high mutagenesis rates

The maternal protein deposition and gene expression of Cas9 in the presence of a *gRNA* transgene were previously reported to induce LOF mutations in some F_1_ progeny from a cross using *nos*- or *vas*-driven Cas9 and U6-gRNA lines (Port *et al.* 2014; Lin and Potter 2016; Oberhofer *et al.* 2018; Kandul *et al.* 2019); however, the somatic nature of F_1_ LOF mutations was not fully explored. This is in part due to the fact that when Cas9 and gRNA are linked together in a single-locus HGD, somatic and germline LOF mutations are not easily distinguishable from heritable mutations occurring in prior generations, which can result in overestimation of mutation rates. Therefore, unlinking these components enables a better method for methodically disentangling these events. Here, using a split-drive design, we were able to carefully assess the effects of timing, expression, and inheritance of Cas9 on both homing and cleavage efficiencies. As reported previously, we found that maternal Cas9 protein deposition was sufficient to induce homing in germ cells, aka. shadow drive (Champer *et al.* 2019c; Guichard *et al.* 2019), in addition to high rates of F_1_ somatic LOF mutations (Port *et al.* 2014; Lin and Potter 2016; Oberhofer *et al.* 2018; Kandul *et al.* 2019). Interestingly, our estimations of homing rates by the shadow drive in *white* locus are notably higher than the previously reported in *yellow* locus: 59–72% (Figure 2) *vs.* 29–32% (Guichard *et al.* 2019) and 38% (Champer *et al.* 2019c), although the ratio of first generation drive to shadow drive frequencies is comparable in these systems (∼50%). The differences may be due to the *yellow* locus being less accessible to cleavage and HDR than *white* locus, or perhaps the lower fitness of *yellow* LOF somatic mutations (see below) biases against their inheritance (Massey *et al.* 2019).

Rather unexpectedly, we found that the zygotic expression of Cas9 alone (paternal Cas9), without the maternally deposited Cas9 protein, was also sufficient to induce F_1_ LOF somatic mutations. In fact, both *nanos* and *vasa* promoters, which were previously characterized to have early germline-limited expression (Van Doren *et al.* 1998; Sano *et al.* 2002; Kondo and Ueda 2013), in our system do support significant somatic expression of Cas9 which may stem from PE or perhaps the use of the P10 3′UTR. For example, F_1_ progeny with both maternally deposited Cas9 protein or with zygotically expressed Cas9 gene inherited from their fathers had *white* and *yellow* LOF somatic mutations with up to 100% efficiency (Figure 2). Consistent observations were reported in a recent work using a *trans*-complementing Gene Drive (tGD) system (Lopez del Amo *et al.* 2019) and for somatically induced lethality of Notch alleles driven by paternally provided Cas9 (Guichard *et al.*, 2019). Taken together, these data conclusively demonstrate that, in the context of the tested promoters, Cas9 somatic expression confounds the estimation of mutagenesis rates in germ cells and can result in the overestimation of homing rates for a single-locus HGD.

### Resistance alleles accumulate over subsequent generations

Consistent with previous studies, we found that maternal deposition of Cas9 protein into embryos inheriting a *GDe* results in both resistance allele formation and homing in the germ cells (Champer *et al.* 2019c; Guichard *et al.* 2019). In addition to this observation, we also found that paternal Cas9 protein deposition was not sufficient to induce mutagenesis in target genes, presumably due to the low quantities of Cas9 carried by the sperm into the egg. Moreover, we determined that maternal deposition of Cas9 protein *in trans*-heterozygous females with *vas*- and nos*-Cas9* does not induce more resistance alleles at the expense of a homing rate than those in the females that inherit *vas*- and nos*-Cas9* paternally.

In the light of substantial somatic expression of Cas9 driven by common *Drosophila* germline promoters (Figure 2), the germline inheritance rate provides a better estimate of the rate of inducing resistance alleles in germ cells than the ‘embryonic resistance allele’ frequency used previously (Champer *et al.* 2019c). Consequently, our estimations of F_2_ resistance allele formation are lower than those reported by Champer *et al.* as the embryo R^2^ (LOF) resistance alleles for a split-gene drive system with the nos*-Cas9*, 11% ± 6% (Figure 2) *vs.* 74% ± 2% (Champer *et al.* 2019c).

The frequency of resistance alleles (*w^R^*) increased dramatically between F_2_ and F_3_ generations and correlated with decreases in homing (Figure 3). Taken together, these results suggest that HDR-mediated homing and NHEJ-mediated formation of resistance alleles are integral outcomes of DSBs repair induced by Cas9/gRNA; and when resistance alleles do not cause lethality or sterility to their carrier, the accumulation of resistance alleles is predicted to impede the spread of the drive (Hammond *et al.* 2017; KaramiNejadRanjbar *et al.* 2018; Oberhofer *et al.* 2018).

### gRNA-mediated effector (GME)

The CRISPR/Cas9 technology was previously used in the combination with multiple gRNAs to knock out or convert different genes simultaneously (*e.g.*, Cong *et al.* 2013; Lopez del Amo *et al.* 2019; Kandul *et al.* 2019; Guichard *et al.* 2019). Here we linked the second gRNA targeting *yellow* to the *GDe* inside the *white* locus, and demonstrated that both *yellow* and *white* were effectively knocked out in heterozygous females. In principle, the GME approach can be used to knock out multiple genes located on different chromosomes, such as multiple host factors required for mosquito infection with pathogens or repressors of mosquito anti-pathogen immune genes (Simões *et al.* 2018). Unlike the the allelic drive (Guichard *et al.* 2019), the GME does not require the HDR-mediated conversion in germ cells; instead, it relies on the NHEJ-mediated *indel* formation in somatic tissues, and the widespread Cas9 somatic expression described here is expected to improve the penetrance of the GME-mediated knockout (Kandul *et al.* 2019). Robust knockout of host genes in somatic tissues may reduce the mosquito fitness (Dong *et al.* 2018), but an efficient gene drive can spread its cargo genes in a population even if they are costly to their carriers (Kyrou *et al.* 2018). Therefore, our results suggest that the GME directing knock out of multiple mosquito genes to suppress pathogen infection in mosquitoes may be a viable strategy and should be further explored going forward.

### Novel strategies for disarming resistance alleles in germ cells

**Figure 4:**
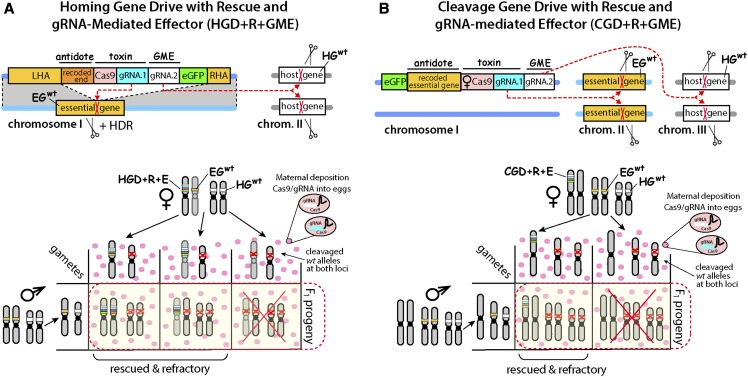
gRNA-mediated effector (GME) incorporated into two novel gene drive designs mechanistically based on lethal biallelic mosaicism. (A) Schematic of Homing Gene Drive targeting an essential gene with a recoded Rescue and GME (HGD+R+GME). The HGD+R+GME expresses Cas9 and two gRNAs targeting an essential gene (EG) and host gene (HG), a marker gene (*eGFP*), and the cleavage-resistant recorded portion of the essential gene that is being targeted by the gRNA/Cas9 complex (Rescue), which can rescue the knockout phenotype, flanked by Left and Right Homology Arms (LHA and RHA). Mechanistically, once HGD+R+GME is integrated precisely inside the EG it will direct cleavage of the EG^wt^ allele on a receiver chromosome, and induce knockout mutations that will either result in lethal biallelic mosaicism, or convert the receiver chromosome into EG^HGD+R+GME^ via homology directed repair (HDR). This ensures that only the progeny that inherit EG^HGD+R+GME^ survive, while all progeny that inherit a cleaved EG allele perish due to non-rescued lethal mosaicism. In addition, the HGD+R+GME induces knockout of HG located on another (or the same) chromosome, leading to desired phenotype (*i.e.*, pathogen resistance) to its carriers. The Punnett square below depicts the genetics of how HGD+R+GME achieves a 100% transmission rate and refractoriness in F_1_ progeny. Female heterozygous for HGD+R+GME maternally deposits Cas9/gRNA complexes into every oocyte knocking out both EG and HG, and only zygotes that inherit the HDR+R+GME would survive as F_1_ progeny. Notably, HDR will convert EG^wt^ alleles into EG^HGD+R+GME^ alleles and further increase numbers of surviving F_1_ progeny and this non-Mendelian inheritance rate will depend on homing efficiencies. (B) Schematic of Cleavage-only Gene Drive targeting an essential gene with a recoded Rescue and GME (CGD+R+GME). The CGD+R+GME expresses Cas9 with multiple gRNAs targeting an ES (gRNA.1) and HG (gRNA.2), a marker gene (*eGFP*), and the cleavage-resistant recorded essential gene (Rescue) integrated at a separate genomic location from the target gene. Mechanistically, a CGD+R+GME drive relies exclusively on cleavage with no HDR required for biased inheritance. A Punnett square depicts the genetics of how CGD+R+GME achieves 100% transmission and infection resistance rates in F_1_ progeny. The female heterozygous for CGD+R+GME deposits Cas9/gRNA complexes into every oocyte, only the half of the zygotes that inherits the CDR+R+MGE in a Mendelian fashion survive as F_1_ progeny, while the other half that do not inherit CDR+R+GME perishes due to lethal biallelic mosaicism.

The accumulation of drive resistance alleles reported here was in part due to the fact that *white* is recessive viable, enabling NHEJ-induced resistance alleles to accumulate. Given this accumulation, targeting non-essential genes using HGD may not be ideal. To avoid this issue, targeting essential genes would be a more appropriate design to ensure gene drive stability and spread. By targeting essential genes, it is possible that non-drive resistance alleles could be actively selected against using a phenomenon previously termed as lethal mosaicism (Kandul *et al.* 2019; Guichard *et al.* 2019) or by natural selection due to increased fitness costs. Lethal mosaicism results in dominant biallelic knockouts of target genes throughout development, which could eliminate cleavage resistance alleles as they would be non-viable. We envision two novel drive design architectures that incorporate a GME and rely on lethal mosaicism to limit the generation of resistance alleles. First, haplo-sufficient genes essential for insect viability or fertility can be targeted by HGD designed to express a recoded version of the disrupted gene that is resistant to gRNA-mediated cleavage in addition to a linked GME (HGD+R+GME). This ensures that only the progeny that inherit the HGD+R+GME survive, while all progeny that inherit a cleaved allele perish due to non-rescued lethal mosaicism (Figure 4). Second, a **C**leavage-only **G**ene **D**rive with **R**escue could be designed that incorporates a GME (CGD+R+GME) which mechanistically relies exclusively on cleavage for biased inheritance and selection against drive resistance alleles (Figure 4)(Oberhofer *et al.* 2019). Both of these strategies would likely be effective in limiting the accumulation of drive resistance alleles. However, in-frame functional mutations (*R1* type) that confer resistance against the Cas9/gRNA and do not cause fitness costs to carriers may still be generated, which could still limit the spread of a drive, and generation of these R1 alleles could possibly be further minimized by inclusion of additional gRNAs that target the essential genes to mediate drive. To summarize, our results demonstrate that inserting a GME into a HGD, efficient knockouts of multiple genes can be achieved while simultaneously biasing GDe transmission rates into subsequent generations. However, resistance alleles were generated, and accumulated, which would limit the efficacy and spread of this system. To overcome these limitations, novel drive architectures are proposed and remain to be tested in future studies.
